# MAPK/ERK Signaling Regulates Insulin Sensitivity to Control Glucose Metabolism in *Drosophila*

**DOI:** 10.1371/journal.pgen.1002429

**Published:** 2011-12-29

**Authors:** Wei Zhang, Barry J. Thompson, Ville Hietakangas, Stephen M. Cohen

**Affiliations:** 1 Institute of Molecular and Cell Biology, Singapore, Singapore; 2 Department of Biological Sciences, National University of Singapore, Singapore, Singapore; 3 London Research Institute, Cancer Research UK, London, United Kingdom; 4 Institute of Biotechnology, University of Helsinki, Helsinki, Finland; University of California San Francisco, United States of America

## Abstract

The insulin/IGF-activated AKT signaling pathway plays a crucial role in regulating tissue growth and metabolism in multicellular animals. Although core components of the pathway are well defined, less is known about mechanisms that adjust the sensitivity of the pathway to extracellular stimuli. In humans, disturbance in insulin sensitivity leads to impaired clearance of glucose from the blood stream, which is a hallmark of diabetes. Here we present the results of a genetic screen in *Drosophila* designed to identify regulators of insulin sensitivity *in vivo*. Components of the MAPK/ERK pathway were identified as modifiers of cellular insulin responsiveness. Insulin resistance was due to downregulation of *insulin-like receptor* gene expression following persistent MAPK/ERK inhibition. The MAPK/ERK pathway acts via the ETS-1 transcription factor Pointed. This mechanism permits physiological adjustment of insulin sensitivity and subsequent maintenance of circulating glucose at appropriate levels.

## Author Summary

Insulin signaling is an important and conserved physiological regulator of growth, metabolism, and longevity in multicellular animals. Disturbance in insulin signaling is common in human metabolic disorders. For example insulin resistance is a hallmark of diabetes and metabolic syndrome. While the core components of the insulin signaling pathway have been well established, the mechanisms that adjust the insulin responsiveness are only known to a limited extent. Here we present results from a genetic screen in *Drosophila* that was designed to identify regulators of cellular insulin sensitivity in an *in vivo* context. Surprisingly, we discovered cross-talk between the epidermal growth factor receptor (EGFR)–activated MAPK/ERK and insulin signaling pathways. This regulatory mechanism, which involves transcriptional control of *insulin-like receptor* gene, is utilized *in vivo* to maintain circulating glucose at appropriate levels. We provide evidence for a regulatory feed-forward mechanism that allows for dynamic transient responsiveness as well as more stable, long-lasting modulation of insulin responsiveness by growth factor receptor signaling. The combination of these mechanisms may contribute to robustness, allowing metabolism to be appropriately responsive to physiological inputs while mitigating the effects of biological noise.

## Introduction

The insulin signaling pathway is a highly conserved regulatory network coordinating animal metabolism and growth with nutritional status. In mammals, energy metabolism is regulated by insulin, and tissue growth by insulin-like growth factors (IGFs), through their respective receptors (for review see [1]). *Drosophila* has a single insulin-like receptor protein (InR), which is activated by a family of insulin-like peptides (ILPs) and mediates physiological responses related to both metabolism and growth (for review see [2]). InR stimulation leads to activation of the phosphatidylinositol 3-kinase (PI3K)/AKT pathway. AKT is recruited to the plasma membrane through phosphatidylinositol-3,4,5-triphosphate (PIP3), which is generated by phosphorylation of PI-4,5-P2 by PI3K (for review see [3]). Membrane-recruited AKT is activated through phosphorylation by PDK1 and by TOR complex 2 [4]–[6]. AKT has several downstream effectors, including FOXO, a Forkhead transcription factor. AKT-mediated phosphorylation promotes retention of FOXO in the cytoplasm, thereby limiting FOXO activity [7]. AKT also promotes the activity of TOR complex 1 by phosphorylating two of its upstream regulators, TSC2 and PRAS40 [8]–[11].

Insulin signaling is involved in homeostatic regulation, gradually adjusting physiological processes in response to variable nutritional conditions. This tuning mode of regulation differs from many developmental signaling pathways, which produce a limited range of outputs (e.g. cell fate). Therefore, it is perhaps not surprising that cellular insulin sensitivity is modulated by input from other signaling pathways. For example, TORC1-regulated S6 kinase (S6K) inhibits expression of the IRS scaffold proteins, which are recruited to activated insulin/IGF receptors, thereby making cells more insulin resistant [12], [13]. Inflammatory signals, on the other hand, are known to cause insulin resistance by c-Jun N-terminal kinase-mediated phosphorylation of the IRS proteins (for review see [14]). This is likely to contribute to the pathogenesis of type 2 diabetes.

While regulation of insulin sensitivity is clearly of physiological importance, identifying novel regulatory mechanisms through genetic screens has been challenging due to the need for a sensitive readout that is also amenable to large-scale screening. The *Drosophila* eye provides such a system. Because insulin signaling limits FOXO activity, overexpression of FOXO can challenge the regulatory capacity of the insulin pathway, creating a sensitized genetic background [6], [15]. Using this strategy to identify *in vivo* modulators of insulin pathway activity, we have uncovered a novel regulatory mechanism influencing insulin sensitivity. We show that the extracellular signal-regulated kinase (ERK)/MAP kinase signaling pathway (for review see [16]) influences cellular insulin responsiveness controlling the expression of the *insulin-like receptor (inr)* gene. This transcriptional regulation is mediated through the Ets-1 orthologue, Pointed, a transcription factor regulated by the MAPK/ERK pathway [17]. This mechanism provides a means for integration of signaling input via the Epidermal growth factor receptor (EGFR)-regulated MAPK/ERK with insulin-like signaling to control systemic glucose homeostasis.

## Results/Discussion

### MAPK/ERK pathway modulates insulin-induced cellular responses

**Figure 1:**
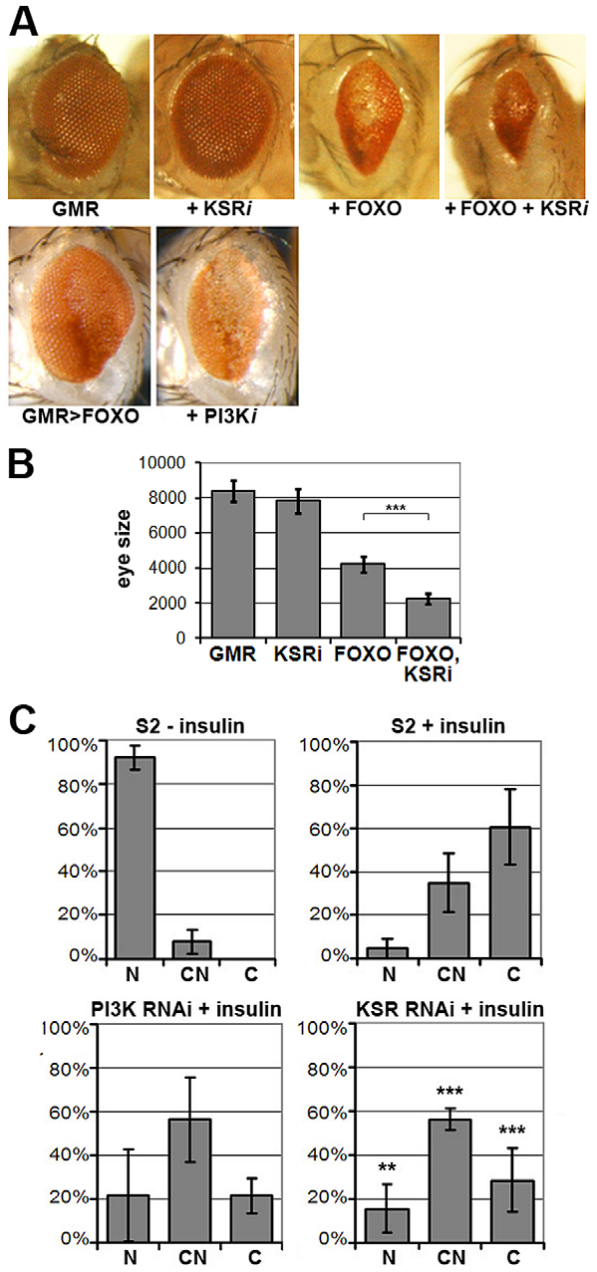
KSR is involved in the regulation of FOXO activity. (A) Photomicrographs of adult eyes. Upper panel: *UAS-KSR^RNAi^* or *UAS-FOXO* or both were expressed in the developing eye with the *GMR-GAL4* driver. Lower panels: *GMR-GAL4+UAS-FOXO* also expressing UAS-RNAi to deplete *PI3K*. (B) Quantification of the total area of affected eyes of the indicated genotypes measured in pixels from digital images using ImageJ. Error bars indicate standard deviation from measurement of at least 6 eyes for each genotype. Student's t-test: (***) p<0.001. (C) Quantification of the subcellular localization of transfected FOXO-GFP. S2 cells with GFP signal were classified into 3 groups according to FOXO localization (N: predominantly nuclear; CN: equal levels in cytoplasm and nucleus; C: predominantly cytoplasmic). Upper panels: compare unstimulated cells with cells stimulated with insulin (10 µg/ml, 30 min). Lower panels: Cells transfected with dsRNA to deplete PI3K or KSR and after 4 days, stimulated with insulin. Error bars represent standard deviation from 3 independent experiments. Fisher's exact test was used to assess the difference between insulin-stimulated S2 cells with and without KSR depletion: (**) p<0.01; (***) p<0.001.

To identify novel modulators of insulin-like signaling we screened for modifiers of FOXO overexpression, which produces small rough eyes. This phenotype has earlier been shown to respond to changes in insulin-like signaling in a highly sensitive manner [6], [15]. As insulin-like signaling is a known regulator of growth, we focused on screening RNAi lines that had earlier shown tissue undergrowth in a wing-based screen ([18], Figure S1). Our screen identified the *PI3K* gene, which serves as a positive control. The screen also identified *kinase suppressor of ras* (*ksr*) as an enhancer of the FOXO gain-of-function phenotype. Downregulation of *ksr* by RNAi enhanced the FOXO phenotype, but on its own, did not reduce eye size (Figure 1A, quantified in Figure 1B). The lack of an obvious eye phenotype resulting from *ksr* depletion alone presumably reflects the magnitude of KSR downregulation generated with the *GMR-GAL4* driver during the phase of eye imaginal disc growth. Enhancement of the FOXO overexpression phenotype was also observed when one copy of the *ksr* gene was removed (Figure S2). Removing one copy of the *ksr* gene on its own did not reduce eye size, indicating the utility of the sensitized background to identify subtle modulators of pathway activity.

FOXO is regulated at multiple levels, including nuclear localization [7]. FOXO is nuclear in cells devoid of growth factors, but upon insulin stimulation FOXO accumulates in the cytoplasm through AKT-mediated phosphorylation ([7]; Figure 1C) As expected, RNAi-mediated depletion of PI3K limited the insulin-induced shift toward cytoplasmic FOXO. Depletion of KSR by RNAi produced a similar effect (Figure 1C; representative images in Figure S3).

**Figure 2:**
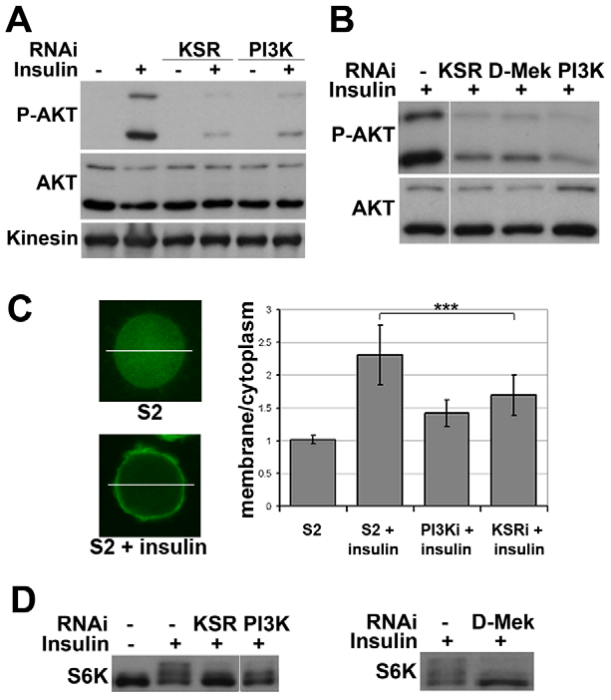
Impaired insulin signaling activation upon MAPK/ERK inhibition. (A) Immunoblots to detect AKT phosphorylation. Cells were treated with dsRNA to deplete KSR or PI3K and stimulated with insulin (10 µg/ml, 30 min; “+”) or left untreated (“−”). AKT phosphorylation was detected by an antibody specific for the phosphorylated form of the AKT ‘hydrophobic motif’ site S505. Antibody to total AKT protein and Kinesin were used as loading controls. (B) Immunoblots to visualize the level of AKT S505 phosphorylation and total AKT in cells treated with dsRNA to deplete KSR, D-MEK or PI3K. Cells were stimulated with insulin (30 min). Samples were run on the same gel, but intervening lanes have been removed as indicated. (C) Visualization of the level of PIP3 in the cell membrane by localization of a GFP-GRP1 PH domain fusion protein. Left panel: photomicrographs showing translocation of GFP-GRP1 PH to the membrane upon insulin stimulation. The ratio of membrane to cytoplasmic GFP levels was measured as pixel intensity along the white line. Right panel: histogram showing the ratio of membrane to cytoplasmic GFP levels. PI3K and KSR depleted cells showed less PH domain membrane localization upon insulin stimulation. Student's t-test: (***) p<0.001. (D) Immunoblots to visualize the level of S6K phosphorylation in cells treated with dsRNA to deplete KSR, PI3K or D-MEK. Cells were stimulated with insulin (10 µg/ml, 30 min). The slower migrating forms correspond to phosphorylated S6K. Left panel: samples were run on the same gel, but intervening lanes have been removed as indicated.

Does KSR act via AKT or does a parallel pathway override AKT-mediated FOXO regulation? To address this, we monitored insulin-induced activation of AKT. Depletion of KSR suppressed insulin-induced phosphorylation of the activating ‘hydrophobic motif’ site S505 on AKT (Figure 2A). The known functions of KSR are related to MAPK/ERK activation [19], [20]. To ask if changes in the canonical MAPK/ERK pathway would explain the KSR effect on insulin signaling, we depleted D-MEK (MAP kinase kinase). This produced an effect comparable to that of KSR (Figure 2B). Insulin-induced AKT activation involves increase in the level of plasma membrane phosphoinositide PIP3 through the activity of PI3K [3]. To assess PIP3 levels, we used a GFP-linked pleckstrin homology (PH) domain from GRP1 [21]. Insulin treatment induced prominent membrane accumulation of the GRP1-PH domain, which was prevented by depletion of PI3K (Figure 2C). Similarly, RNAi-mediated depletion of KSR reduced membrane localization of GRP1-PH in response to insulin.

To test whether other downstream targets of AKT besides FOXO were affected by MAPK/ERK inhibition, we analyzed phosphorylation of S6K, a target of TORC1 [22]. Insulin-induced phosphorylation shifts some of the S6K protein into a ladder of slower migrating forms [e.g. [23]], which was reduced by depletion of KSR or D-MEK (Figure 2D). In both experiments the effects were comparable to depletion of PI3K. To test, whether MAPK/ERK signaling affected PI3K activity independent of insulin, we overexpressed activated PI3K in the absence of insulin stimulation and monitored AKT phosphorylation. In this setting, knockdown of KSR had no influence on pathway activity (Figure S4). In sum, these data suggest that reduced MAPK/ERK activity lowers sensitivity to insulin stimulation, but does not hamper PI3K from activating AKT.

### MAPK/ERK regulates *inr* gene expression via the ETS-1 transcription factor Pointed

**Figure 3:**
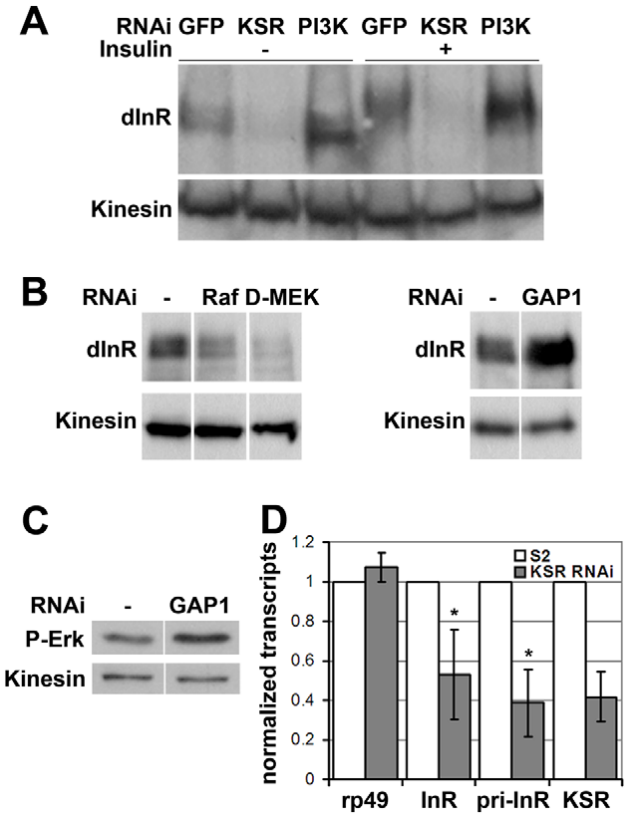
MAPK/ERK signaling regulates *inr* gene expression. (A) Immunoblot to visualize the level of InR protein. Cells were treated with dsRNA to deplete PI3K, KSR or GFP as a control and after 5 days stimulated with insulin. In control cells, insulin stimulation results in a phosphorylation-induced mobility shift in SDS-PAGE. Anti-Kinesin was used as loading control. (B) Immunoblots to visualize the level of InR protein in cells treated with dsRNA to deplete Raf, D-MEK or GAP1. Samples were run on the same gel, but intervening lanes have been removed as indicated. (C) Immunoblot to visualize the MAPK/ERK activity using an antibody specific to the phosphorylated form of ERK. S2 cells were treated with dsRNA to deplete GAP1 or left untreated. Anti-Kinesin was used as a loading control. Samples were run on the same gel, but intervening lanes have been removed as indicated. (D) Histogram showing the levels of *rp49*, *inr* and *ksr* mRNAs measured by quantitative RT-PCR. S2 cells were treated with dsRNA to deplete *ksr* (gray bars) or left untreated (white bars). Total RNA were extracted and normalized for cDNA synthesis. RNA levels were normalized to *kinesin* mRNA. The efficiency of *ksr* depletion was ∼60%. *inr* denotes the mature mRNA; *pri-inr* denotes the unspliced nuclear primary transcript measured using intron-specific primers. Error bars represent standard deviation from 3 independent experiments. Student's t-test: (*) p<0.05.

The data above suggested that MAPK signaling regulates the insulin-like pathway above the level of PI3K. We therefore sought to monitor InR expression level as well as activation making use of a shift in its electrophoretic mobility caused by phosphorylation (Figure 3A, lanes 1 and 4). Surprisingly, we found that depletion of KSR led to a reduction in the total level of InR protein. This was observed in both insulin-treated and untreated cells. Depletion of PI3K did not produce a comparable effect. To confirm that InR expression is regulated by the canonical MAPK/ERK pathway, we silenced the expression of Raf and D-MEK, which also showed reduced InR expression (Figure 3B). Increasing MAPK/ERK pathway activity acts in the opposite direction: depletion of Gap1, the GTPase activator of RAS, led to activation of MAPK/ERK signaling visualized by phospho-specific antibody against the active form of ERK (Figure 3C) as well as elevated InR expression (Figure 3B). Thus, regulation of InR appears to be a specific MAPK/ERK pathway effect and InR levels are sensitive to both positive and negative changes in the MAPK/ERK activity.

**Figure 4:**
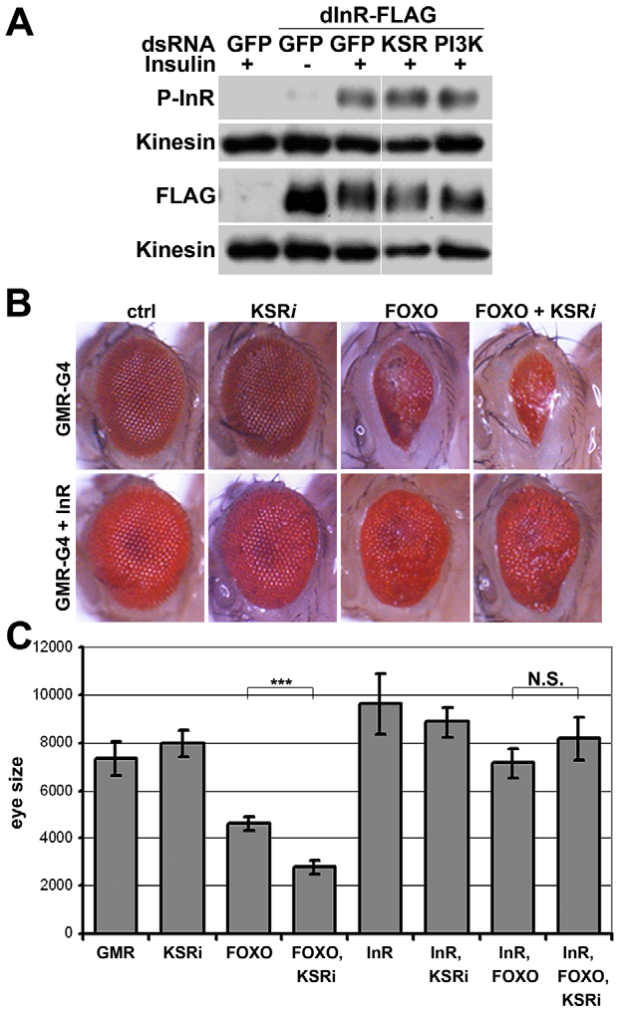
InR transgene expressed under heterologous promoter is insensitive to KSR depletion. (A) Immunoblots to visualize the level of Flag-tagged InR protein expressed under control of the pMT promoter in transfected S2 cells. Cells were treated with dsRNA to deplete PI3K, KSR or GFP as a control and after 5 days stimulated with insulin. Insulin activity was visualized using antibody specific to the phosphorylated form of InR (upper panel). Total Flag-InR was visualized with anti-Flag. Anti-Kinesin was used as loading control. Samples were run on the same gel, but intervening lanes have been removed as indicated. (B) Adult eyes expressing *GMR-Gal4* and *UAS-FOXO*, *UAS-InR* and/or a *UAS-ksr^RNAi^* transgene to deplete KSR. (C) Quantification of the total eye area measured in pixels from digital images using ImageJ. Error bars indicate standard deviation from measurement of at least 5 eyes for each genotype. Student's t-test: (***) p<0.001. (N.S.) indicates that there was no significant effect of KSR depletion in the *UAS-FOXO+UAS-InR* eyes.

How does MAPK/ERK signaling affect InR expression? Using quantitative RT-PCR we observed a significant reduction in the levels of the mature *inr* mRNA and the unspliced *inr* primary transcript upon KSR depletion in S2 cells (Figure 3D). The reduction in *inr* primary transcript levels upon KSR depletion suggests that MAPK/ERK activity is likely to regulate *inr* transcription. If so, we would expect transgene-directed expression of *inr* to be refractory to the effects of MAPK pathway modulation. This proved to be the case. A transfected version of *inr* under control of a heterologous promoter was insensitive to KSR depletion (Figure 4A). As an *in vivo* test we used *GMR-Gal4* as a heterologous promoter to direct expression of a *UAS-InR* transgene in the eye. Under these conditions, KSR depletion did not enhance the FOXO overexpression phenotype (Figure 4B, 4C). The insensitivity of transgene-directed InR to the effects of KSR depletion is consistent with the hypothesis of a mechanism involving control of endogenous *inr* transcription.

**Figure 5:**
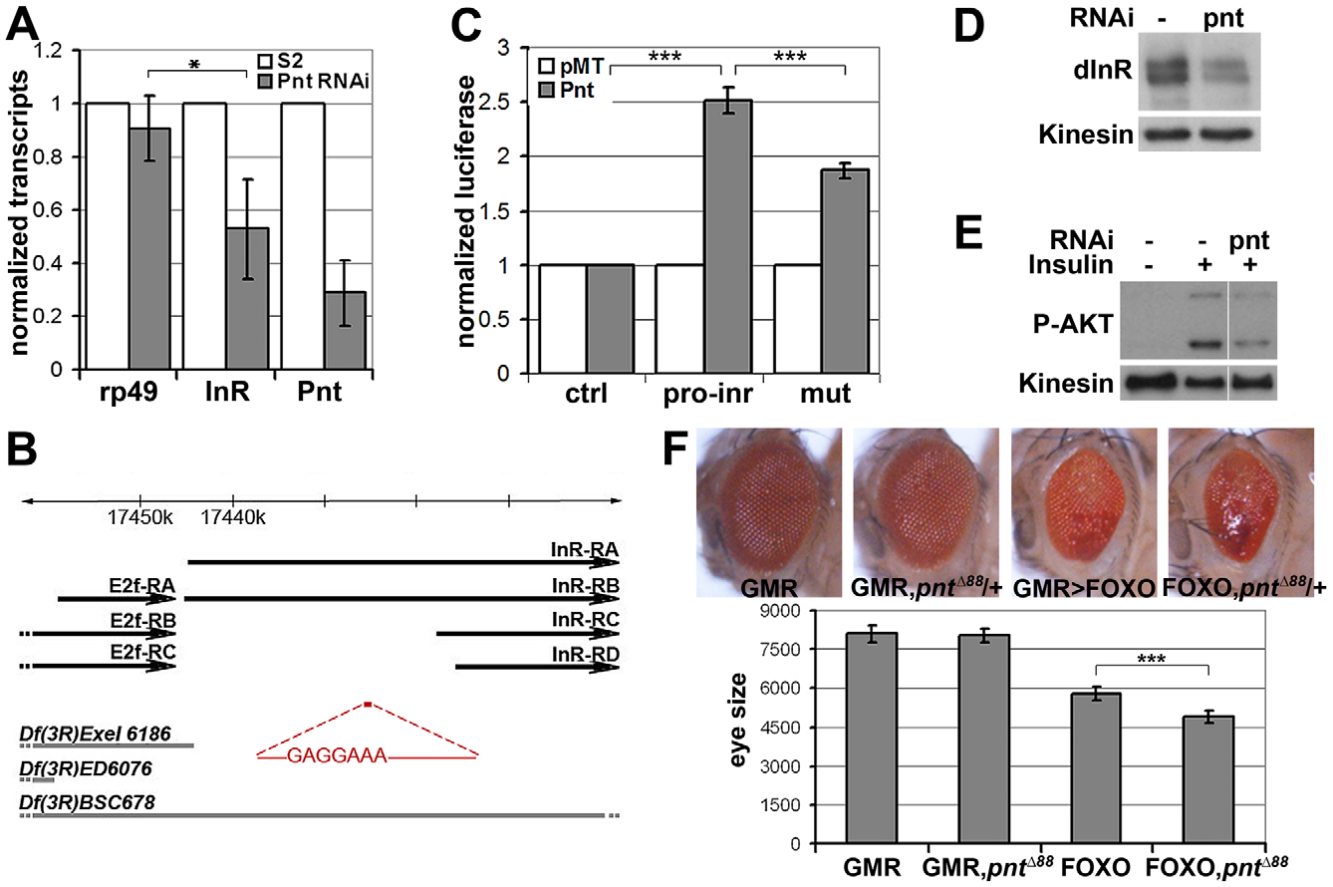
*inr* is regulated by the ETS-1 transcription factor Pointed. (A) Measurement of *inr* transcript levels in S2 cells transfected with dsRNA to deplete *pointed* mRNA or left untreated. The efficiency of *pointed* depletion was ∼70%. *inr* was reduced to ∼50% compared to *rp49* after normalization to *kinesin* mRNA. Error bars represent standard deviation based on 4 independent experiments. Student's t-test: (*) p<0.05. (B) Schematic representation of the *inr* locus. Thick black arrow lines indicate transcripts of *inr* and *E2F*. The *cis*-regulatory region identified by luciferase assay is shown in red and the Pnt consensus site sequence is indicated. Thick gray lines represent deletions used in Figure 7 as well as Figures S7 and S8. Note that the deletions affect multiple genes. Only *inr* and adjacent *E2F* loci are indicated. (C) Luciferase assays showing activation of the reporter plasmids after cotransfection with a vector to express Pnt-P2 (gray bars) or empty vector as a control (white bars). pro-inr denotes luciferase reporter with the DNA element from the *inr cis* control region shown in (B). mut denotes luciferase reporter with the Pnt consensus site mutated. pGL3-Basic was used as the control reporter. Error bars represent standard deviation based on 4 independent experiments. Student's t-test: (***) p<0.001. (D) Immunoblot to visualize the level of InR protein in S2 cells treated with dsRNA to deplete *pointed* or left untreated. Anti-Kinesin was used as a loading control. (E) Immunoblot to visualize the level of AKT S505 phosphorylation in S2 cells treated with dsRNA to deplete *pointed*. After 5 days cells were stimulated with insulin (30 min). (F) Upper panel: Photographs of adult eyes. From left to right: *GMR-Gal4* alone; GMR-Gal4 with one mutant copy of the *pointed* gene (*pnt^Δ88^* allele); *GMR-Gal4+UAS-FOXO*; *GMR-Gal4+UAS-FOXO* with one mutant copy of the *pointed* gene (*pnt^Δ88^* allele). Lower panel: Plot of total eye area measured in pixels from digital images using ImageJ. Error bars indicate standard deviation from measurement of at least 5 eyes for each genotype. Student's t-test: (***) p<0.001.

*pointed* (*pnt*) encodes an ETS-1 transcription factor that is activated by MAPK/ERK through phosphorylation of a conserved threonine residue, T151 [24], [25]. We tested its involvement in the regulation of *inr* expression by depletion of Pointed from S2 cells by RNAi. This caused a prominent reduction of *inr* mRNA (Figure 5A). To narrow down the regulatory region of the *inr* gene we systematically analyzed >20 kb upstream of the *inr* coding region by preparing a series of luciferase reporter constructs (Figure S5), which lead to identification of a minimal *cis*-regulatory region of 0.8 kB (Figure 5B). Overexpression of Pointed-P2 in S2 cells increased reporter activity directed by this 0.8 kB region (Figure 5C). This fragment contained one site perfectly matching the consensus Pointed binding site (5′-(C/G)(A/C/G)GGA(A/T)(A/G)-3′; [26]). Mutating the consensus site reduced the ability of Pointed to induce reporter expression (Figure 5C), suggesting binding to this site contributes to Pointed-mediated regulation of *inr* gene expression. Reciprocally, Pointed depletion in S2 cells led to a decrease in the level of InR protein (Figure 5D), and to a reduction of insulin-induced AKT S505 phosphorylation (Figure 5E).

To further test this relationship *in vivo*, we asked whether reducing *pointed* levels would influence the severity of the FOXO overexpression phenotype. Removing one copy of the *pointed* gene using three independent alleles modestly but significantly enhanced the FOXO overexpression phenotype in the eye (Figure 5F, Figure S6). These findings suggest that the MAPK/ERK pathway acts via the Ets-1 transcription factor Pointed to control cellular insulin sensitivity.

### MAPK/ERK regulates *inr* gene expression to control glucose metabolism

**Figure 6:**
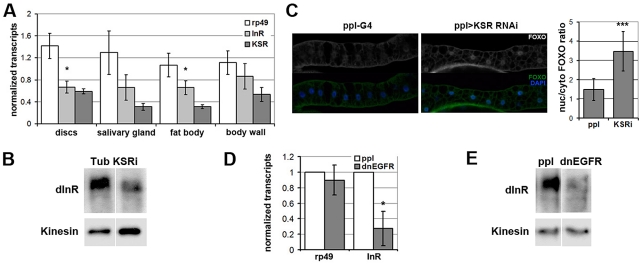
EGFR-MAPK/ERK signaling regulates InR expression and FOXO localization *in vivo*. (A) Histogram showing the levels of *rp49*, *inr* and *ksr* mRNAs measured by quantitative RT-PCR. Wandering 3^rd^ instar larvae expressed *UAS-ksr^RNAi^* under ubiquitous *tubulin-Gal4* control. Controls expressed *tubulin-Gal4* without the UAS-RNAi transgene. Total RNA was isolated from imaginal discs, fat body, salivary gland and body wall. RNA levels were normalized for cDNA synthesis before Q-PCR. RNA levels were normalized to *kinesin* mRNA. The efficiency of KSR depletion is shown in dark gray bars. Student's t-test: (*) p<0.05. (B) Immunoblot to detect the level of InR protein in fat body from wandering 3^rd^ instar larvae expressed *UAS-KSR^RNAi^* under *Tubulin-Gal4* control. Samples were run on the same gel, but intervening lanes have been removed as indicated. (C) Left panels: Immunofluorescent images of fat body dissected from wandering 3^rd^ instar larvae stained with anti-FOXO (green). Nuclei were labeled with DAPI (Blue). Larvae expressed *UAS-ksr^RNAi^* under *pumpless-Gal4* control. Control larvae expressed Gal4 without the RNAi transgene. Right panel: Quantification of the ratio between nuclear FOXO and cytoplasmic FOXO in fat body expressing *ppl-Gal4* or with *UAS-ksr^RNAi^*. Subcellular regions were defined by DAPI staining. FOXO intensities were measured in pixels from digital images using ImageJ. Error bars represent standard deviation from measurement of at least 24 cells for each genotype. Student's t-test: (***) p<0.001. (D) Histogram showing the levels of *rp49* and *inr* mRNAs measured by quantitative RT-PCR. Wandering 3^rd^ instar larvae expressed *UAS-dnEGFR* under *pumpless-Gal4* control. Controls expressed Gal4 without the UAS transgene. Total RNA was isolated from the fat body. RNA levels were normalized to *kinesin* mRNA. Error bars represent standard deviation from 3 independent experiments. Student's t-test: (*) p<0.05. (E) Immunoblot to visualize the level of InR protein in fat body from wandering 3^rd^ instar larvae expressing *UAS-dnEGFR* under *pumpless-Gal4* control. Samples were run on the same gel, but intervening lanes have been removed as indicated.

To begin to explore the physiological role of regulation of InR levels by the MAPK/ERK pathway *in vivo*, we performed a survey of larval tissues and found that KSR depletion led to significant reduction of *inr* transcript levels in imaginal discs and larval fat body, *Drosophila* equivalent of liver and adipose tissue (Figure 6A). KSR depletion also led to a reduction of InR protein levels (Figure 6B) as well as nuclear FOXO accumulation in the larval fat body (Figure 6C). MAPK/ERK signaling can be regulated by a variety of receptor tyrosine kinases (RTKs). We next made use of *pumpless-GAL4* to manipulate RTK activity. Pumpless is active mainly in the fat body, but also other tissues, such as parts of larval gut. Inhibition of Epidermal growth factor (EGF) signaling by expression of a dominant negative form of EGFR (dnEGFR) led to downregulation of *inr* mRNA (Figure 6D) and protein levels (Figure 6E) in the isolated fat body. This suggests that EGFR is a physiologically relevant upstream regulator of MAPK/ERK-mediated control in Inr expression *in vivo*.

**Figure 7:**
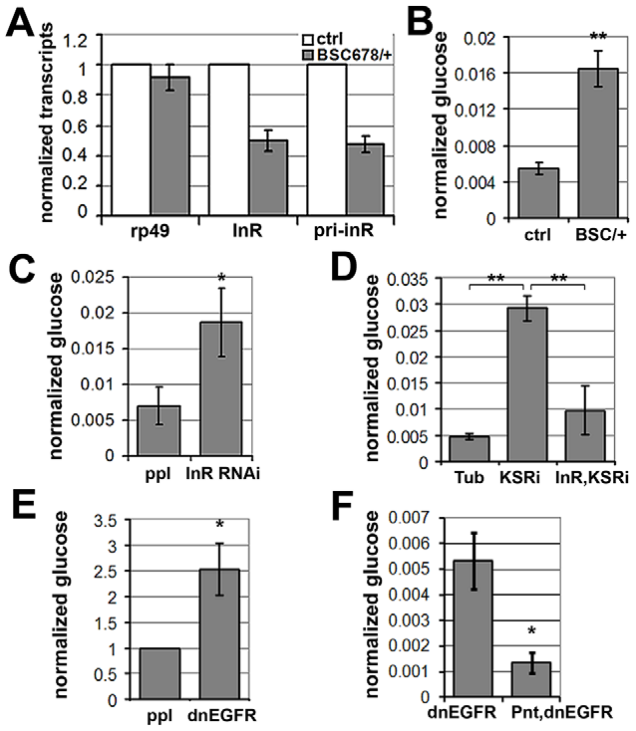
MAPK/ERK regulates *inr* expression to control circulating glucose levels. (A) Histogram showing the levels of *rp49*, *inr* mRNAs and *inr* primary transcript (*pri-inr*) measured by quantitative RT-PCR in control (*y^−^, w^−^*) and *Df(3R)BSC678/+* larvae. Error bars represent standard deviation from 3 independent experiments. (B) Histogram showing glucose levels in control (*y^−^, w^−^*) and *Df(3R)BSC678/+* larval hemolymph. Error bars represent standard deviation from 3 independent experiments. Student's t-test: (**) p<0.01. (C) Histogram showing glucose levels in hemolymph from larvae expressing *UAS-InR^RNAi^* under *pumpless-Gal4* control. Error bars represent standard deviation from 3 independent experiments. Student's t-test: (*) p<0.05. (D) Histogram showing the levels of circulating glucose in hemolymph. Wandering 3^rd^ instar larvae expressed *UAS-KSR^RNAi^* under *Tubulin-Gal4* with or without co-expressed *UAS-inr*. Controls expressed Gal4 without the RNAi transgenes. Student's t-test: (**) p<0.01. (E) Histogram showing glucose levels in hemolymph from larvae expressing *UAS-dnEGFR* under *pumpless-Gal4* control. Error bars represent standard deviation from 3 independent experiments. Student's t-test: (*) p<0.05. (F) Histogram showing the levels of circulating glucose from larvae expressing *UAS-dnEGFR* without or with overexpression of Pointed under *pumpless-Gal4* control. Error bars represent standard deviation from 3 independent experiments. Student's t-test: (*) p<0.05.

Systemic regulation of InR activity has been shown to influence metabolic homeostasis [reviewed in [27]]. In this context, the effects of KSR depletion on *inr* expression and FOXO localization in fat body were suggestive of a link to energy metabolism. To ask whether reduction of *inr* to half of normal levels was sufficient to cause a metabolic disturbance, we made use of larvae carrying one copy of the deletion *Df(3R)BSC678*, which fully removes the *inr* gene (Figure 5B). Quantitative RT-PCR was used to confirm that *inr* mRNA levels were reduced to ∼50% in these animals (Figure 7A). Notably, this result indicates that there is little or no feedback from InR signaling on *inr* expression, and suggests that there is limited output from InR via the MAPK pathway in *Drosophila*. In addition, we observed that flies with modestly reduced *inr* levels showed impaired capacity to limit FOXO activity in the eye (Figure S7).

Larvae lacking one copy of the *inr* gene showed no significant change in levels of stored glycogen and triglycerides or trehalose (Figure S8), a circulating disaccharide synthesized by the fat body through glycogenolysis ([28]). However, levels of circulating glucose in the hemolymph were substantially increased (Figure 7B), suggesting compromised clearance of dietary glucose from the circulation. To ask whether this phenotype could be achieved by independent genetic means, we used *pumpless*-*GAL4* to drive expression of a *UAS-inr^RNAi^* transgene to deplete InR (Figure 7C). These animals showed elevated glucose in their hemolymph, verifying that regulation of InR levels is physiologically important *in vivo* in maintaining levels of circulating glucose. This conclusion was further supported by the finding that InR overexpression modestly, but significantly, decreased levels of circulating glucose (Figure S9). These findings are consistent with what has been reported in flies in which the ability to produce insulin-like peptides was compromised by genetic ablation of the insulin-producing neurosecretory cells [29]. These flies showed a prominent metabolic change at the level of circulating glucose levels [29]. Similarly, insulin signaling in mammals regulates glucose uptake and reduced insulin sensitivity is linked to hyperglycemia, metabolic syndrome and type-2 diabetes [30], [31].

To test whether MAPK/ERK-regulation of InR expression is involved in maintaining systemic glucose homeostasis, we assessed the effects of KSR RNAi. Depletion of KSR led to elevated levels of circulating glucose (Figure 7D). If the effect of KSR depletion is due to reduced InR levels, we would expect restoring *inr* expression under Gal4 control to lower glucose levels toward normal. This proved to be the case (Figure 7D). As expected, expression of a dnEGFR using *pumpless-GAL4* driver also resulted in elevated circulating glucose (Figure 7E). The glucose levels were restored by simultaneous overexpression of Pointed, which is in agreement with the view that Pointed acts as a downstream effector of the pathway (Figure 7F). These observations suggest a physiological role for EGFR-MAPK/ERK-Pointed activity in control of glucose homeostasis via regulation of InR levels.

The insulin signal transduction pathway is regulated by cross-talk from several other signaling pathways. This includes input from the amino-acid sensing TOR pathway into regulation of insulin pathway activity by way of S6 kinase regulating IRS [12]–[14]. Signaling downstream of growth factor receptors has also been linked to regulation of insulin signaling [32], [33]. The active form of the small GTPase Ras can bind to the catalytic subunit of PI3K and promote its activity. Expression of a form of PI3K that cannot bind Ras allows insulin signaling, but at reduced levels [33]. The work reported here provides evidence for a second mechanism through which growth factor receptor signaling through the MAPK/ERK pathway modulates insulin pathway activity. Transcriptional control of *inr* gene expression by EGFR signaling may provide a means to link developmental signaling to regulation of metabolism. In this context, we noted a statistically significant correlation between EGFR target gene *sprouty* and *inr* gene expression at different stages during *Drosophila* development (Figure S10).

Several steps of the insulin pathway can be regulated by phosphorylation. Given that the MAPK/ERK pathway is a kinase cascade, a priori, the possibility of phosphorylation-based interaction between these pathways would seem likely. However, this appears not to be the case. Acute pharmacological inhibition of the MAPK/ERK pathway proved to have no impact on insulin pathway activity (Figure S11). Thus short-term changes in MAPK/ERK pathway activity do not seem to be used for transient modulation of insulin pathway activity. Instead, the MAPK/ERK pathway acts through the ETS-1 type transcription factor Pointed to control expression of the *inr* gene. Transcriptional control of *inr* suggests a slower, less labile influence of the MAPK pathway. Taken together with the earlier studies [32], [33], our findings suggest that growth factor signaling can regulate insulin sensitivity by both transient and long-lasting mechanisms.

Why use both short-term and long-term mechanisms to modulate insulin responsiveness to growth factor signaling? The use of direct and indirect mechanisms that elicit a similar outcome is reminiscent of feed-forward network motifs [34]. Although these motifs are often thought of in the context of transcriptional networks, the properties that they confer are also relevant in the context of more complex systems involving signal transduction pathways. In multicellular organisms, feed-forward motifs are often used to make cell fate decisions robust to environmental noise [35]. Our findings suggest a scenario in which a feed-forward motif is used in the context of metabolic control, linking growth factor signaling to insulin responsiveness. In this scenario, growth factor signaling acts directly via RAS to control PI3K activity and indirectly via transcription of the *inr* gene to elicit a common outcome – sensitization of the cell to insulin. This arrangement allows for a rapid onset of enhanced insulin sensitization, followed by a more stable long-lasting change in responsiveness. Thus a transient signal can both allow for an immediate as well as a sustained response. The transcriptional response also makes the system stable to transient decreases in steady-state growth factor activity. We speculate that this combination of sensitivity and stability allows responsiveness while mitigating the effects of noise resulting from the intrinsically labile nature of RTK signaling. As illustrated by our data, failure of this regulation in the fat body leads to elevated circulating glucose levels, likely reflecting impaired clearance of dietary glucose from the circulation by the fat body. Maintaining circulating free glucose levels low is likely to be important due to the toxic effects of glucose [28]. In contrast, circulating trehalose, glycogen or triglyceride levels showed no significant change in animals with reduced InR expression, suggesting that these aspects of energy metabolism can be maintained through compensatory mechanisms in conditions of moderately impaired insulin signaling.

Earlier studies by Puig and coworkers have shown that the transcription of the *inr* gene is under dynamic control [36], [37]. Activation of FOXO in the context of low insulin signaling leads to upregulation of *inr* transcription, thus constituting a feedback regulatory loop. Thus, InR expression appears to be under control of two receptor-activated cues, which have opposing activities: *inr* expression is positively regulated by the EGFR-MAPK/ERK module, but negatively regulated by its own activity on FOXO. In the setting of this study, the cross-regulatory input from the MAPK/ERK pathway was found to dominate over the autoregulatory FOXO-dependent mechanism. If conditions exist in which the FOXO-dependent mechanism was dominant, we would expect to observe a limited potential for crossregulation by the MAPK/ERK pathway. Whether Pointed and FOXO display regulatory cooperativity at the *inr* promoter is an intriguing question for future study.

## Materials and Methods

### Fly strains

*UAS-InR*, *pnt^Δ88^*, *pnt^07825^*, *UAS-Pnt-P2*, *ksr^S-627^*, *Df(3R)Exel6186*, *Df(3R)ED6076* and *Df(3R)BSC678* flies were obtained from the Bloomington Stock Center. *UAS-RNAi-PI3K*, *UAS-RNAi-KSR* and *UAS-RNAi-InR* lines were from the Vienna *Drosophila* RNAi center. *pUAST-FOXO-GFP* flies were provided by Aurelio Teleman. *pUAST-dnEGFR* flies were provided by Pernille Rørth. *pnt^T5^* flies were provided by Christian Klämbt.

### Cell culture and treatments

S2 cells were grown at 25°C in SFM (Gibco) supplemented with L-glutamine. dsRNA was prepared using MegascriptT7 (Ambion) with the following templates: *PI3K*, nucleotides 358–857 of Pi3K92E coding sequence (FBpp0083348); *ksr*, nt 2224–2710 (FBpp0078413); *D-MEK*, nt 961–1191 of the ORF plus the first 83 nt of the 3′UTR (FBtr0071313); *Raf*, nt 522–912 (FBpp0110324);*GAP1*, nt 153–646 (FBpp0076096); *pnt*, nt 1541–1957 (718AA isoform, FBpp0088658); GFP, nt 17–633 of EGFP2, was used as control. S2 cells were treated with 37 nM dsRNA. Cells were transfected using effectene reagent (QIAGEN) with *pMT-GAL4*, *pUAST-FOXO-GFP* or *pUAST-Myc-Dp110CAAX*, or *pMT-GFP-PH* or *pMT-InR-Flag* or *pMT-Pnt-P2*. 0.7 mM CuSO4 was used to induce FOXO, Dp110, GFP-PH, InR or Pnt expression after transfection. The following primers were used to clone InR-Flag into pMT vector with EcoRI, NotI and XhoI sites by fusion: 5′-GGTACCTACTAGTCCAGTGTGGTGGAATTCATGTTCAATATGCCACGGGGAGTGAC-3′; 5′- TTCGAAGGGCCCTCTAGACTCGAGCGGCCGCTTACTTGTCATCGTCGTCCTTGTAGTCCGCCTCCCTTCCGATGAATCCA-3′; 5′- ACGTTGCGCTCGAGCCAGAGCTCGA-3′ and 5′- TCGAGCTCTGGCTCGAGCGCAACGT-3′. The primers used to clone pntp2 into pMT-Myc by SLIC at EcoRI site were: forward, 5′-AGTGCAACTAAAGGGGAATTCATGGAATTGGCGATTTGTAAAACAG-3′; reverse, 5′- GATAAGCTTCTGCTCGAATTCATCCACATCTTTTTTCTCAATCTTAAG-3′. The primers used to clone the *inr* gene regulatory region into pGL3-Basic at HindIII and XhoI sites were: forward, 5′- GCGTGCTAGCCCGGGCTCGAGTGAGAGTTTCATGTGTCAGA -3′; reverse, 5′- AAGCTTACTTAGATCGCAGATGTTAATTGCACAGCAAGCTC-3′. The primers used to mutate the predicted Pnt consensus site with QuickChange II XL kit were: forward, 5′-GAGAATGCCGGAGATGAAGACGCGAACGAAGATGAAGTCGATG-3′; reverse, 5′- CATCGACTTCATCTTCGTTCGCGTCTTCATCTCCGGCATTCTC-3′.

### Cell imaging

For FOXO-GFP localization, live S2 cells were imaged using a Leica SP5 confocal microscope. Images were taken of random fields within 15 min after 10 µg/ml insulin boost for 30 min and scored for GFP localization (scoring was done ‘blind’). For GFP-PH images were taken within 10 min after 10 µg/ml insulin boost for 5 min. The ratio of membrane to cytoplasmic GFP levels was measured as pixel intensity along the white line as indicated in Figure 2C (left panel). For fat body FOXO immunofluorescent staining, newly hatched 1^st^ instar larvae were seeded at 50/vial and reared at 25°C. Wandering 3^rd^ instar larvae were dissected. Tissues were fixed in PBS with 4% paraformaldehyde at room temperature for 20 min. Anti-FOXO antibody [36] was used at 1∶1000 dilution. Fat body connected with salivary gland was imaged using a Zeiss LSM700 confocal microscope.

### Immunoblotting

Cells were homogenized in SDS sample buffer, boiled and resolved by SDS-PAGE before transfer to nitrocellulose membranes for antibody labeling. Antibodies to phospho-S505-AKT, AKT, P-InR and Myc were from Cell Signaling Technology. Anti-Kinesin was from Cytoskeleton. Phospho-ERK antibody was from Sigma. Anti-S6K is described in [38]. Anti-dInR is described in [37].

### Quantitative RT–PCR

Total RNA was extracted from S2 cells using QIAGEN RNeasy Mini Kit and treated with On-Column DNase (QIAGEN RNase-Free DNase Set) at room temperature for 15 min to eliminate genomic DNA contamination. Reverse transcription to synthesize the first strand used oligo-dT primers and Superscript RT-III (Invitrogen). PCR was performed using POWER SYBR GREEN Master Mix (Applied Biosystems) and analyzed on Applied Biosystems 7500 fast real-time PCR system. Results were normalized to *Kinesin* mRNA, and *rp49* was used as a control. The following primers were used: Kinesin-f, 5′-GCTGGACTTCGGTCGTAGAG-3′; Kinesin-r, 5′- CTTTTCATAGCGTCGCTTCC-3′; rp49-f, 5′- GCTAAGCTGTCGCACAAA-3′; rp49-r, 5′- TCCGGTGGGCAGCATGTG-3′; InR-f, 5′- CTGGTGGTGCTGACAGAGAA-3′; InR-r, 5′- GCAGCTGACAACTGGCATTA-3′; pri-InR-f, 5′- CAAGAGACAGCAACAAAAGG-3′; pri-InR-r, 5′- GCTTGCATGTGTTGGTGAGC-3′; KSR-f, 5′- AGCCGAGCGAAGATTGTAAA-3′; KSR-r, 5′- TCCCGATACATGCCTACACA-3′; pnt-f, 5′- CGATGCGAATGCCTACTACACG-3′; pnt-r, 5′- TGCTGGTGTTGTAGCCTGAAC-3′.

### Metabolic analysis

Newly hatched 1^st^ instar larvae were seeded at 50/vial and reared at 25°C. Hemolymph was extracted from wandering stage 3^rd^ instar larvae. 2 µl of pooled hemolymph was diluted with 8 µl Tris buffered saline (pH 6.6) and incubated at 70°C for 5 min before clarification by centrifugation at 20 000×g for 1 min. Glucose was measured in 6 µl supernatant using the GAGO-20 kit (Sigma) and normalized to the same amount of TBS as blank control. For trehalose measurement, supernatant was incubated with 7.5 µg trehalase (Sigma) overnight at 37°C and measured using GAGO-20 kit as well. For glycogen and triglyceride measurements, 3^rd^ instar larvae were homogenized using Sartorius Potter-S tissue homogenizer in water with 0.05% Tween. Supernatant was collected after 5 min of heat inactivation at 70°C and centrifugation at 13000 rpm for 3 min. Glycogen and protein levels were measured using Glycogen assay kit (Bio Vision) and Bio-Rad protein assay reagent, respectively. Triglyceride was measured using Sigma Triglyceride kit. Data were normalized to total protein.

## Supporting Information

Figure S1 Effect of depleting KSR, AKT or Raf on growth in the wing. UAS RNAi transgenes targeting AKT, KSR or Raf were expressed in the posterior compartment of the wing imaginal discs under control of engrailed-Gal4. Knockdown of MAPK/ERK pathway components lead to undergrowth similar to that caused by suppression of insulin signaling pathway. Upper panels: show photographs of the resulting adult wings. Red dots indicate the border between anterior and posterior compartments. Note the reduced area of the P compartment. Lower panels: higher magnification views showing individual cell size (each cell produces a single hair, so cell size can be inferred from the spacing of the hairs).
(TIF)

Figure S2 Effect of reduced *ksr* gene dosage on the FOXO overexpression phenotype. Upper panel: Photographs of adult eyes. From left to right: *GMR-Gal4* alone; *GMR-Gal4* with one mutant copy of the *ksr* gene; *GMR-Gal4+UAS-FOXO*; *GMR-Gal4+UAS-FOXO* with one mutant copy of the *ksr* gene. Lower panel: Plot of total eye area measured in pixels from digital images using Image J. Error bars indicate standard deviation from measurement of at least 5 eyes for each genotype. (*) Student's t-test for removing one copy of *ksr* in *GMR-Gal4+UAS-FOXO* eyes p<0.05.
(TIF)

Figure S3 Representative images showing FOXO localization in S2 cells. Subcellular localization of FOXO-GFP in S2 cells visualized by confocal microscopy. Left panel: (N) predominantly nuclear. Middle panel: (CN) equal levels in cytoplasm and nucleus. Right panel: (C) predominantly cytoplasmic.
(TIF)

Figure S4 KSR acts upstream of PI3K. Immunoblots to visualize the level of AKT and ERK phosphorylation in S2 cells transfected to express the membrane-tethered form of Dp110 (CAAX) compared to control cells transfected with the empty vector. Cells were cultured in serum-free medium and were not stimulated by addition of insulin. Upper to lower: antibody to phosphorylated S505 AKT; antibody to total AKT; Antibody to the phosphorylated form of ERK; antibody to the Myc epitope tag to visualize expression of the Dp110 transgene. Samples were run on the same gel, but intervening lanes have been removed as indicated.
(TIF)

Figure S5 Schematic representation of the fragments used to locate the *inr cis*-regulatory region. Thick black arrow lines indicate transcripts of *inr*. The five putative *cis*-regulatory regions analyzed by luciferase assay are shown as horizontal lines below. The thick line indicates the active region, which was used to narrow down to the 0.8 kb element in Figure 5B. Note that only *inr* gene is indicated.
(TIF)

Figure S6 Independent *pointed* alleles modestly but significantly enhanced the FOXO overexpression phenotype in the eye. (A) Adult eyes expressing *GMR-Gal4* and *UAS-FOXO* without or with one mutant copy of the *pointed* gene (*pnt^07825^* or *pnt^T5^* as indicated). (B) Plot of total eye area measured in pixels from digital images using ImageJ. Error bars indicate standard deviation from measurement of 7 eyes for each genotype. Student's t-test: (***) p<0.001.
(TIF)

Figure S7 Genetic tests of reduced *inr* function *in vivo*. (A,B) Adult eyes expressing *GMR-Gal4* alone or with *UAS-FOXO*. *Exe/+* indicates flies with one copy of the deletion *Df(3R)Exel6186* that partially removes *inr* locus. The total area of eyes was measured in pixels from digital images using ImageJ. Error bars indicate standard deviation from measurement of at least 5 eyes for each genotype. Student's t-test: (***) p<0.001. (C) Histogram showing the level of *inr* mRNA measured by quantitative RT-PCR in control and *Df(3R)Exel6186*/+ flies. (D) Control deletion *Df(3R)ED6076* does not sensitize to FOXO overexpression. Adult eyes expressing *GMR-Gal4* alone or with *UAS-FOXO*. +/+ indicates 2 intact copies of the *inr* locus. *Df(3R)ED6076/+* indicates the flies with one copy of the deletion, which is otherwise similar to *Df(3R)Exel6186*, but does not affect *inr*. Histograms showing quantification of eye size of the indicated genotypes are shown at right. Error bars indicate standard deviation from measurement of at least 5 eyes for each genotype. See Figure 5B for schematic representation of the deletions used to disrupt *inr* function.
(TIF)

Figure S8 No change in levels of stored glycogen and triglycerides or circulating trehalose. Histograms showing glycogen (left) and triglyceride (right) levels normalized to total protein in control (+/+) and *Df(3R)BSC678/+* larvae. Error bars represent standard deviation from 3 independent experiments. Lower panel: Histogram showing trehalose levels in hemolymph from wandering 3^rd^ instar control (+/+) and *Df(3R)BSC678/+* larvae.
(TIF)

Figure S9 InR overexpression modestly, but significantly, decreased levels of circulating glucose. Wandering 3^rd^ instar larvae expressed *UAS-inr* under *Tubulin-Gal4* control. Error bars represent standard deviation from at least 3 independent experiments. Student's t-test: (*) p<0.05.
(TIF)

Figure S10 Correlation between the levels of *inr* and EGFR target *sty* mRNAs at various stages of *Drosophila* development. RNA levels were determined using RNA-seq data from modENCODE (www.modencode.org) by cufflinks (cufflinks.cbcb.umd.edu). Spearman's correlation coefficient rho = 0.71 (P<0.001). E# indicates hours of embryonic development. L# indicates larval stage. P# indicates days of pupal development. ad5d indicates 5 day adult.
(TIF)

Figure S11 Acute pharmacological inhibition of MAPK/ERK has no impact on insulin activity. Immunoblots to visualize the level of AKT and ERK phosphorylation in S2 cells treated with the MEK inhibitor U0126 (Promega) or in control cells. Cells were treated with dsRNA to deplete PI3K, KSR or GFP as a control and after 5 days stimulated with insulin and treated with 10 µM U0126 for 10 min as indicated. Pharmacological inhibition of MEK by U0126 was effective, as visualized by anti-P-ERK. Anti-Kinesin was used as loading control. Samples were run on the same gel, but intervening lanes have been removed as indicated.
(TIF)
