# Supplementary figures and images for: MAPK/ERK Signaling Regulates Insulin Sensitivity to Control Glucose Metabolism in Drosophila
Source: PLoS Genet. 2011 Dec 29;7(12):e1002429. doi: 10.1371/journal.pgen.1002429 (PMC3248469; doi:10.1371/journal.pgen.1002429)

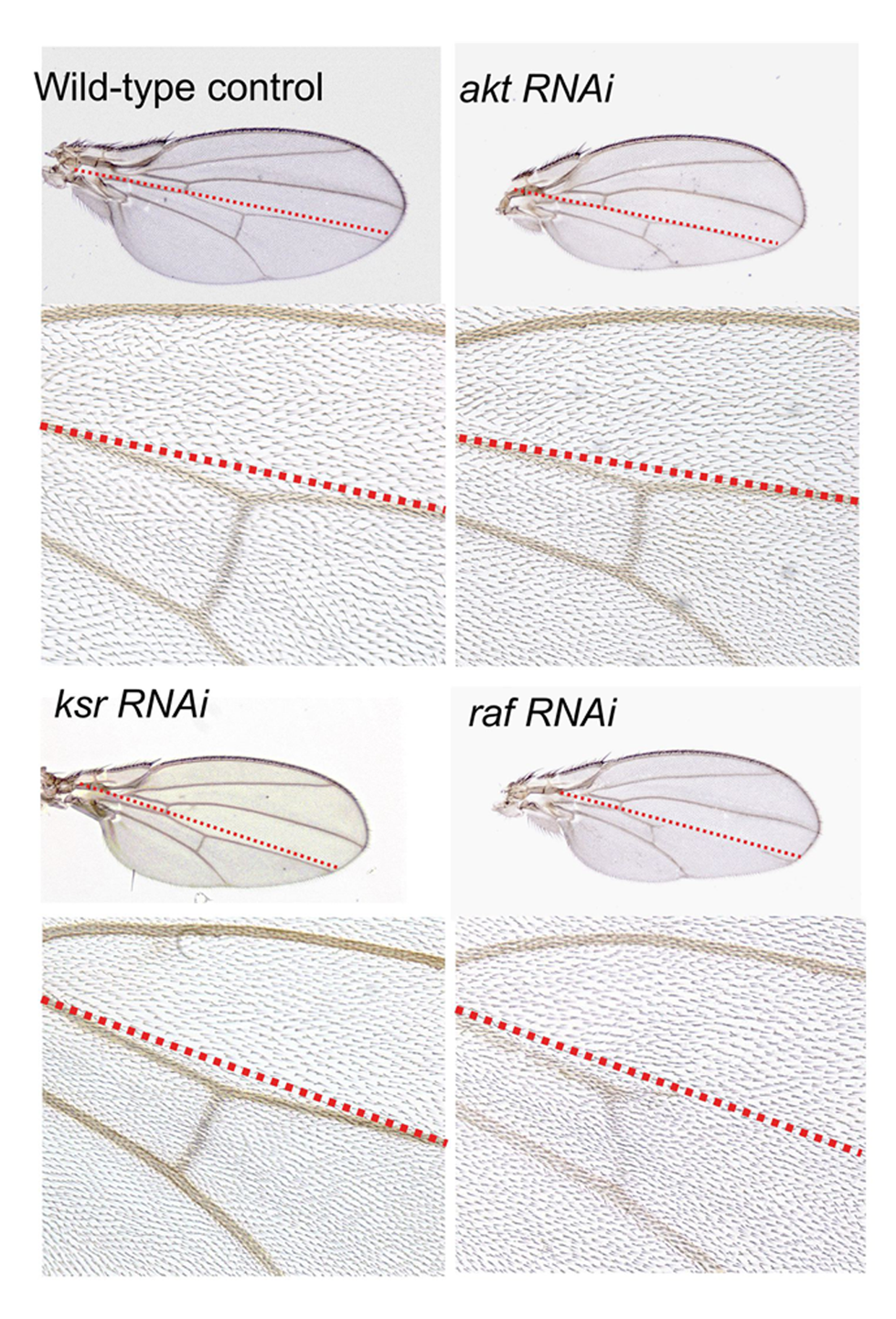

Supplement: Figure S1 — Effect of depleting KSR, AKT or Raf on growth in the wing. UAS RNAi transgenes targeting AKT, KSR or Raf were expressed in the posterior compartment of the wing imaginal discs under control of engrailed-Gal4. Knockdown of MAPK/ERK pathway components lead to undergrowth similar to that caused by suppression of insulin signaling pathway. Upper panels: show photographs of the resulting adult wings. Red dots indicate the border between anterior and posterior compartments. Note the reduced area of the P compartment. Lower panels: higher magnification views showing individual cell size (each cell produces a single hair, so cell size can be inferred from the spacing of the hairs). (TIF) [file pgen.1002429.s001.tif]

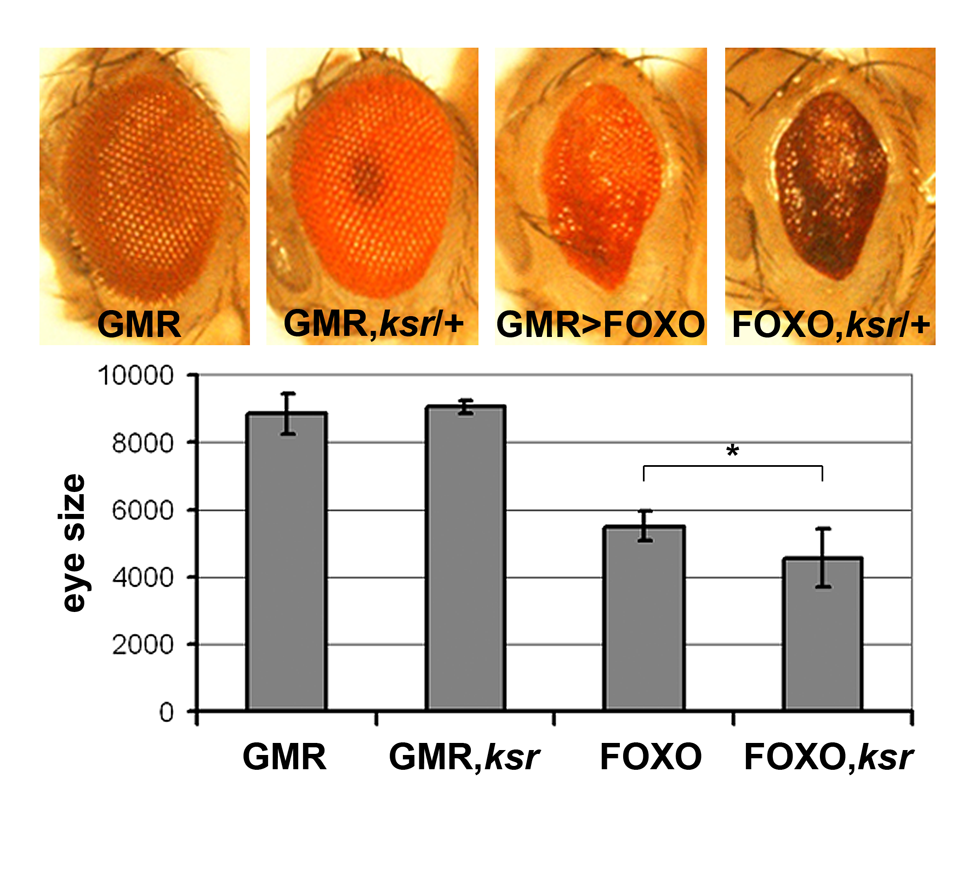

Supplement: Figure S2 — Effect of reduced ksr gene dosage on the FOXO overexpression phenotype. Upper panel: Photographs of adult eyes. From left to right: GMR-Gal4 alone; GMR-Gal4 with one mutant copy of the ksr gene; GMR-Gal4+UAS-FOXO; GMR-Gal4+UAS-FOXO with one mutant copy of the ksr gene. Lower panel: Plot of total eye area measured in pixels from digital images using Image J. Error bars indicate standard deviation from measurement of at least 5 eyes for each genotype. (*) Student's t-test for removing one copy of ksr in GMR-Gal4+UAS-FOXO eyes p<0.05. (TIF) [file pgen.1002429.s002.tif]

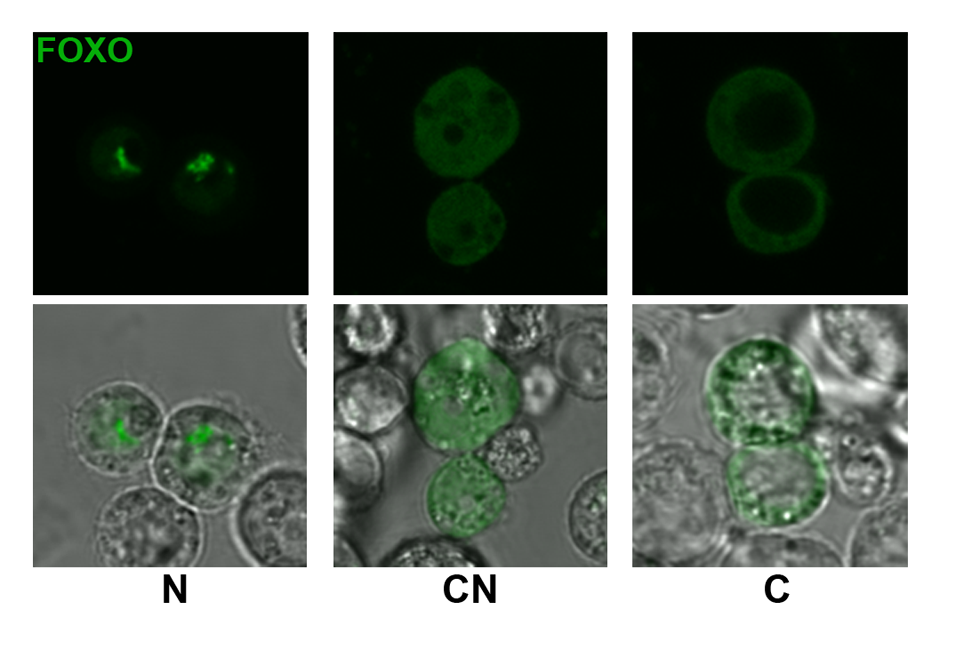

Supplement: Figure S3 — Representative images showing FOXO localization in S2 cells. Subcellular localization of FOXO-GFP in S2 cells visualized by confocal microscopy. Left panel: (N) predominantly nuclear. Middle panel: (CN) equal levels in cytoplasm and nucleus. Right panel: (C) predominantly cytoplasmic. (TIF) [file pgen.1002429.s003.tif]

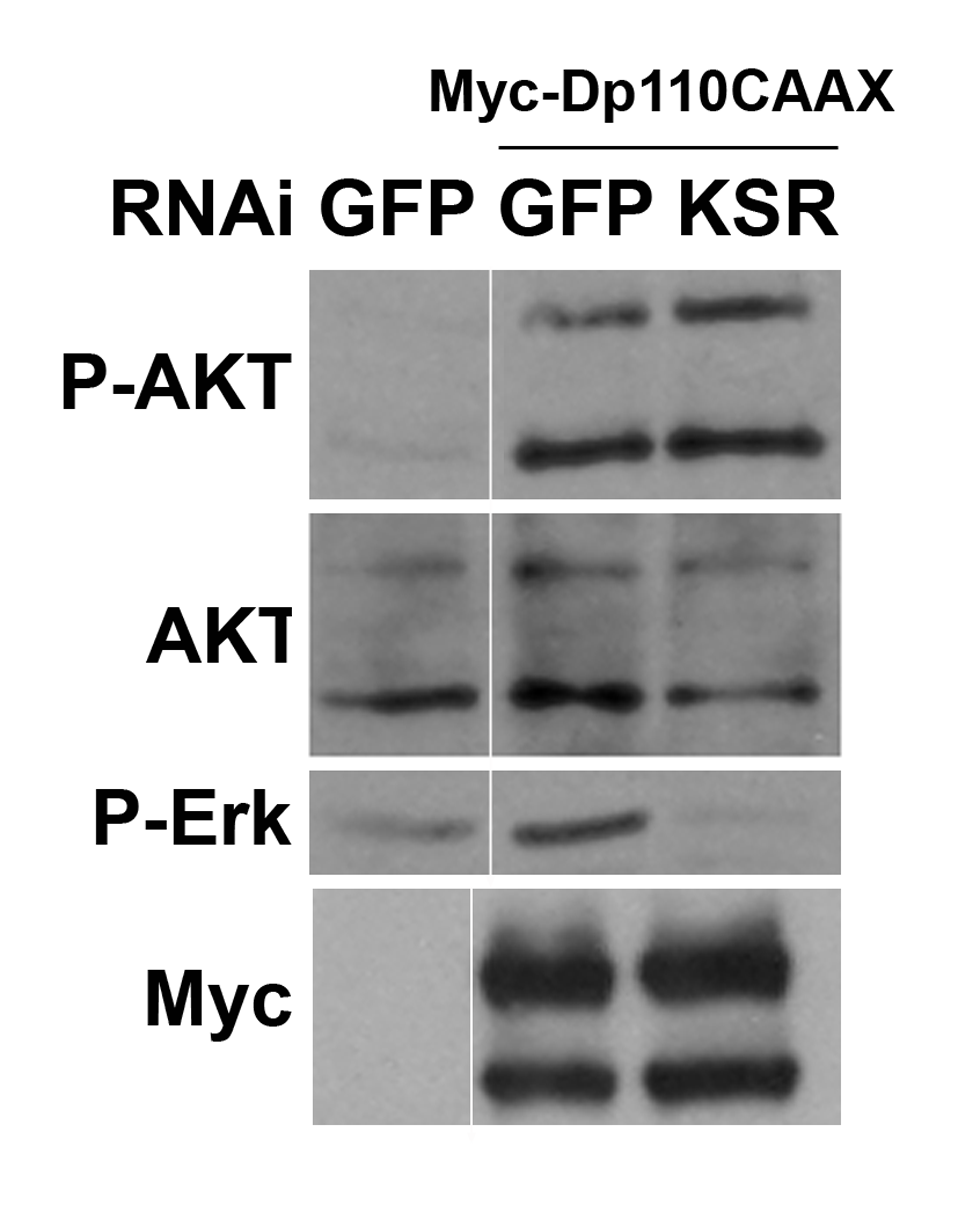

Supplement: Figure S4 — KSR acts upstream of PI3K. Immunoblots to visualize the level of AKT and ERK phosphorylation in S2 cells transfected to express the membrane-tethered form of Dp110 (CAAX) compared to control cells transfected with the empty vector. Cells were cultured in serum-free medium and were not stimulated by addition of insulin. Upper to lower: antibody to phosphorylated S505 AKT; antibody to total AKT; Antibody to the phosphorylated form of ERK; antibody to the Myc epitope tag to visualize expression of the Dp110 transgene. Samples were run on the same gel, but intervening lanes have been removed as indicated. (TIF) [file pgen.1002429.s004.tif]

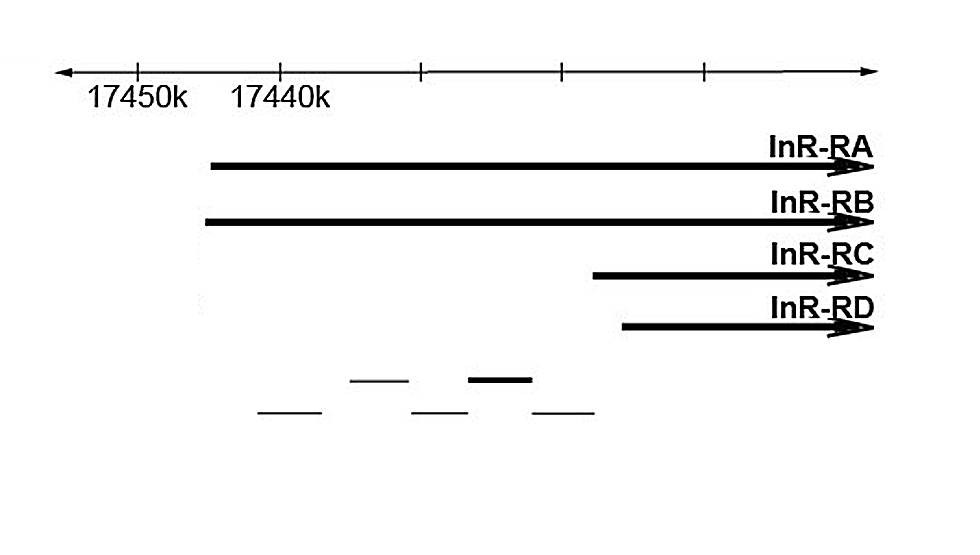

Supplement: Figure S5 — Schematic representation of the fragments used to locate the inr cis-regulatory region. Thick black arrow lines indicate transcripts of inr. The five putative cis-regulatory regions analyzed by luciferase assay are shown as horizontal lines below. The thick line indicates the active region, which was used to narrow down to the 0.8 kb element in Figure 5B. Note that only inr gene is indicated. (TIF) [file pgen.1002429.s005.tif]

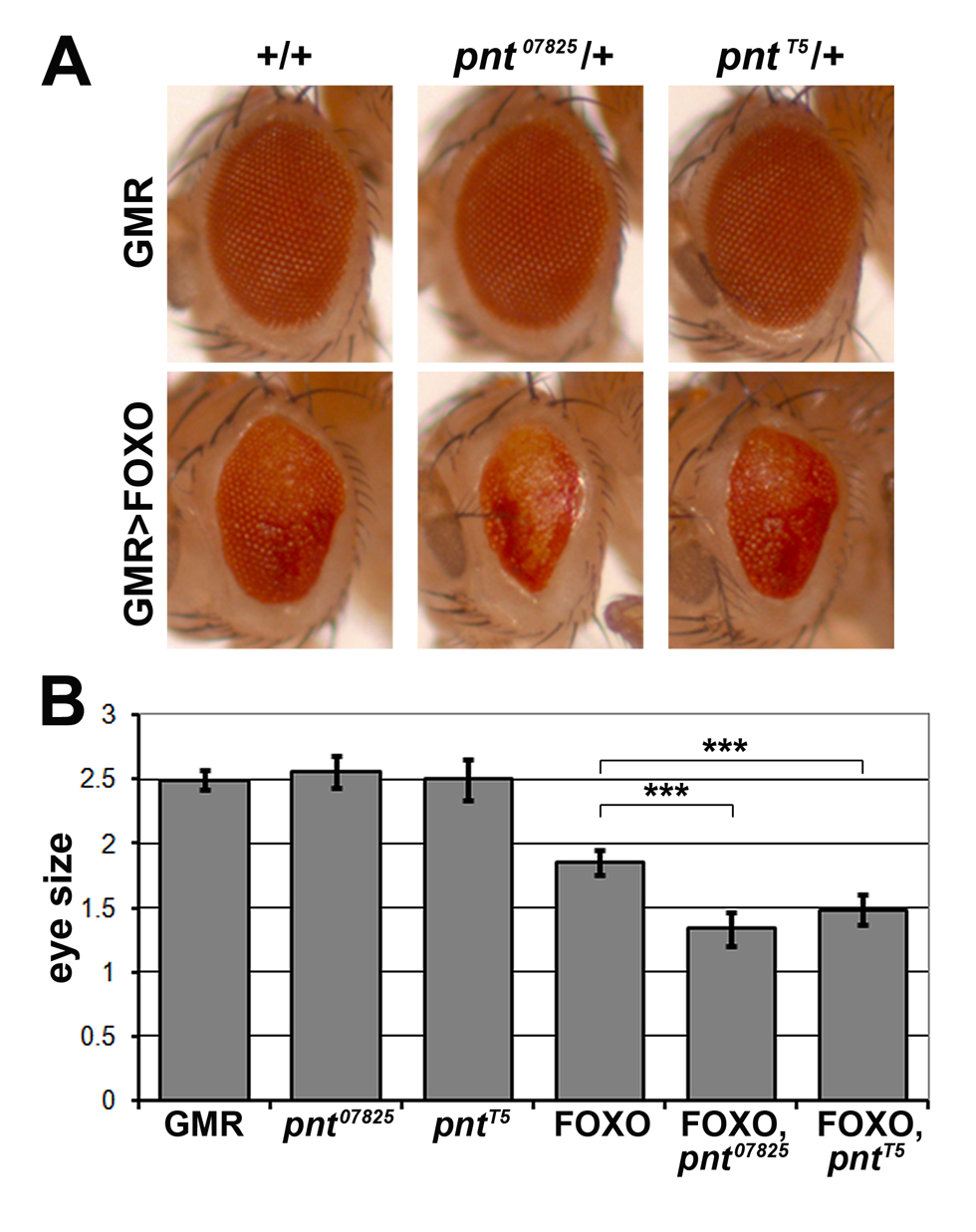

Supplement: Figure S6 — Independent pointed alleles modestly but significantly enhanced the FOXO overexpression phenotype in the eye. (A) Adult eyes expressing GMR-Gal4 and UAS-FOXO without or with one mutant copy of the pointed gene (pnt07825 or pntT5 as indicated). (B) Plot of total eye area measured in pixels from digital images using ImageJ. Error bars indicate standard deviation from measurement of 7 eyes for each genotype. Student's t-test: (***) p<0.001. (TIF) [file pgen.1002429.s006.tif]

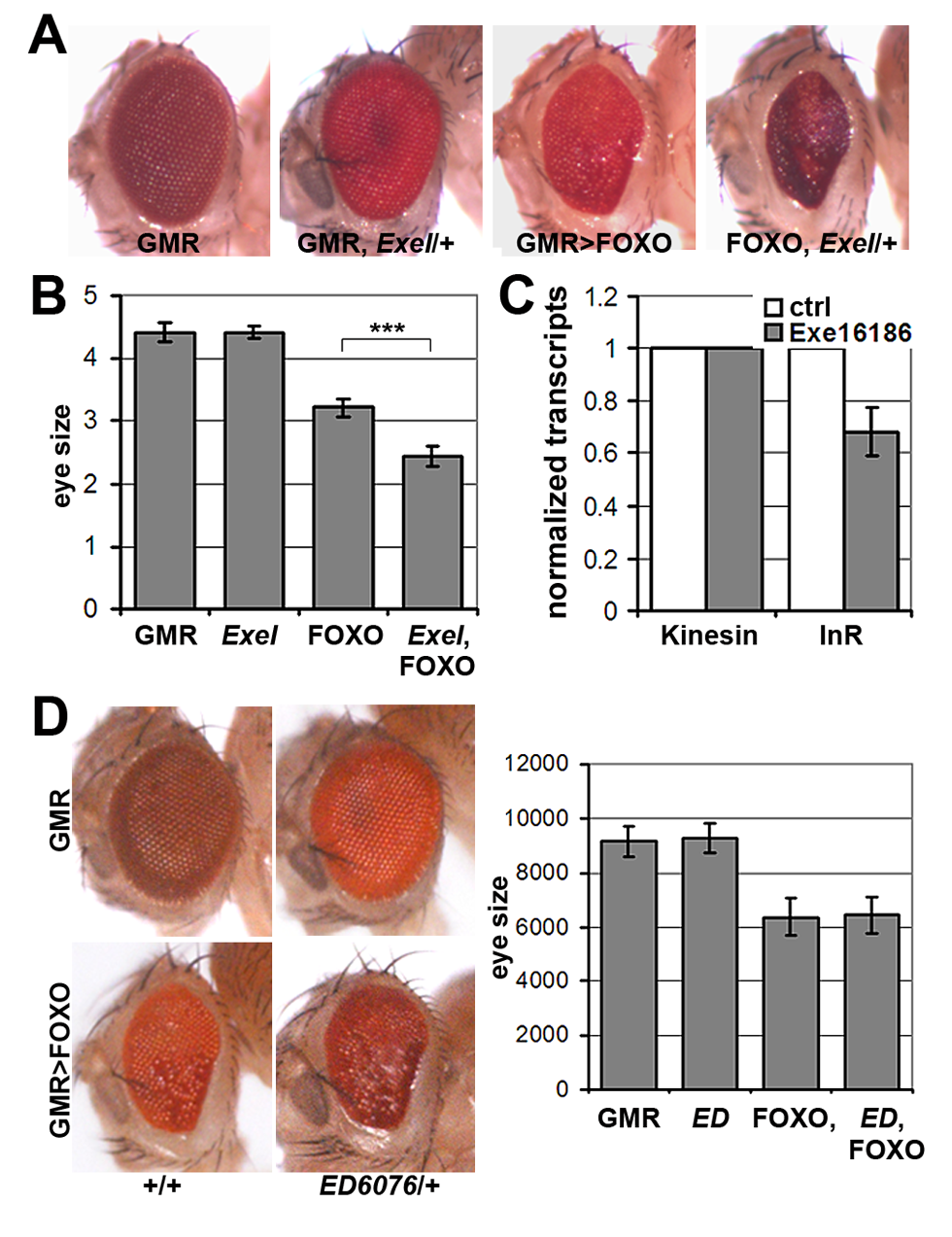

Supplement: Figure S7 — Genetic tests of reduced inr function in vivo. (A,B) Adult eyes expressing GMR-Gal4 alone or with UAS-FOXO. Exe/+ indicates flies with one copy of the deletion Df(3R)Exel6186 that partially removes inr locus. The total area of eyes was measured in pixels from digital images using ImageJ. Error bars indicate standard deviation from measurement of at least 5 eyes for each genotype. Student's t-test: (***) p<0.001. (C) Histogram showing the level of inr mRNA measured by quantitative RT-PCR in control and Df(3R)Exel6186/+ flies. (D) Control deletion Df(3R)ED6076 does not sensitize to FOXO overexpression. Adult eyes expressing GMR-Gal4 alone or with UAS-FOXO. +/+ indicates 2 intact copies of the inr locus. Df(3R)ED6076/+ indicates the flies with one copy of the deletion, which is otherwise similar to Df(3R)Exel6186, but does not affect inr. Histograms showing quantification of eye size of the indicated genotypes are shown at right. Error bars indicate standard deviation from measurement of at least 5 eyes for each genotype. See Figure 5B for schematic representation of the deletions used to disrupt inr function. (TIF) [file pgen.1002429.s007.tif]

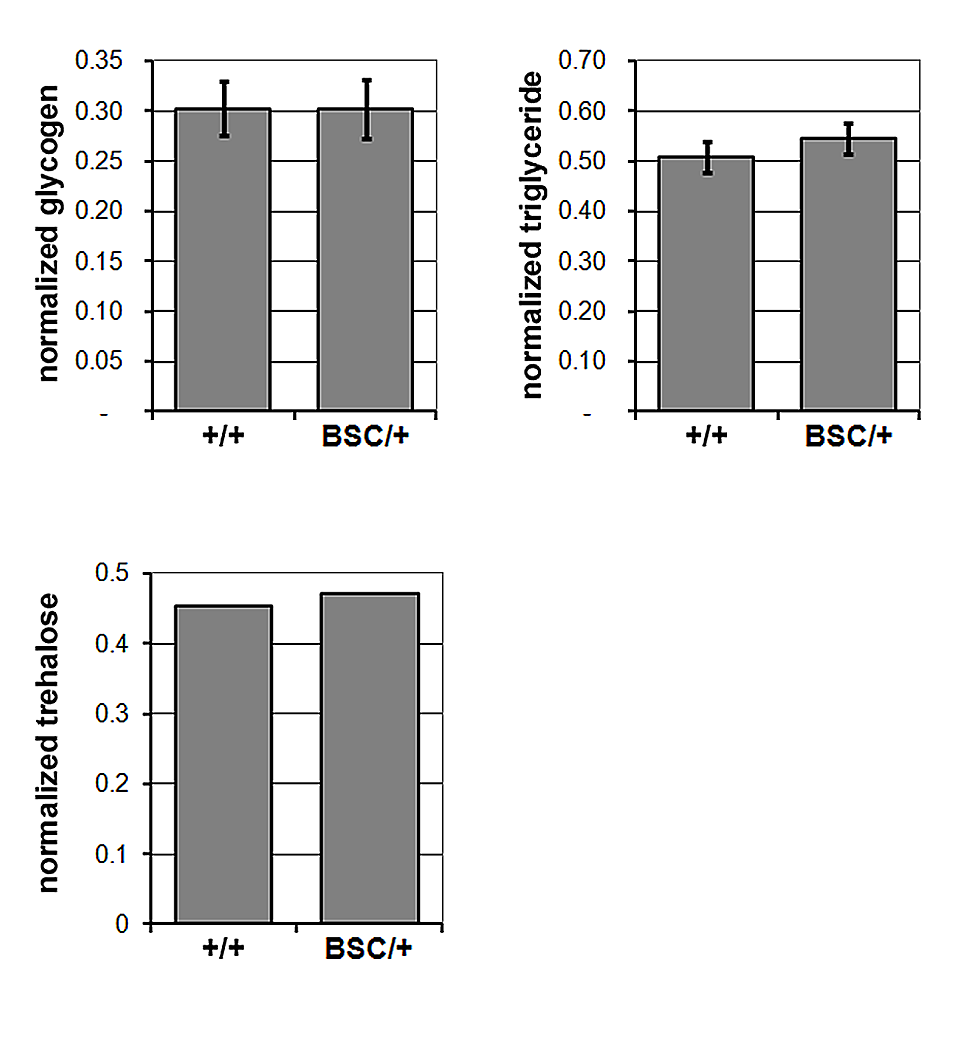

Supplement: Figure S8 — No change in levels of stored glycogen and triglycerides or circulating trehalose. Histograms showing glycogen (left) and triglyceride (right) levels normalized to total protein in control (+/+) and Df(3R)BSC678/+ larvae. Error bars represent standard deviation from 3 independent experiments. Lower panel: Histogram showing trehalose levels in hemolymph from wandering 3rd instar control (+/+) and Df(3R)BSC678/+ larvae. (TIF) [file pgen.1002429.s008.tif]

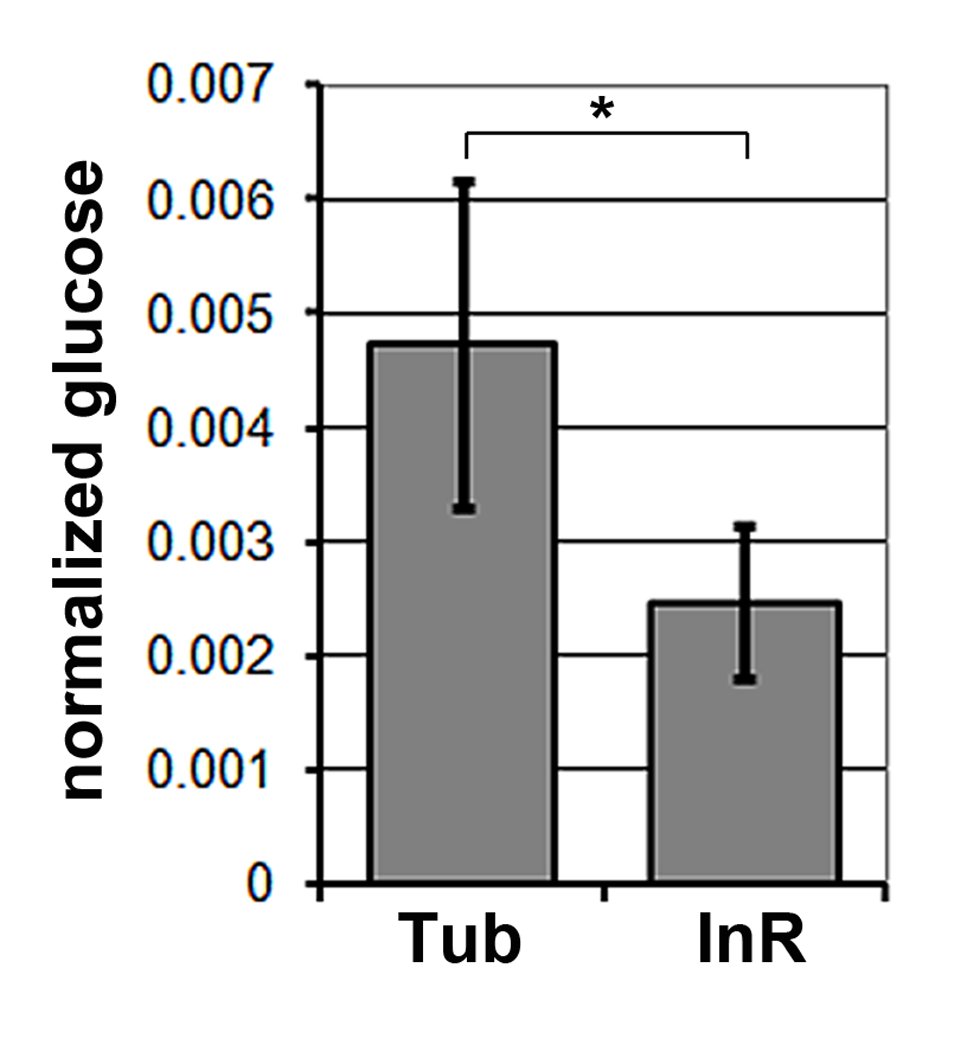

Supplement: Figure S9 — InR overexpression modestly, but significantly, decreased levels of circulating glucose. Wandering 3rd instar larvae expressed UAS-inr under Tubulin-Gal4 control. Error bars represent standard deviation from at least 3 independent experiments. Student's t-test: (*) p<0.05. (TIF) [file pgen.1002429.s009.tif]

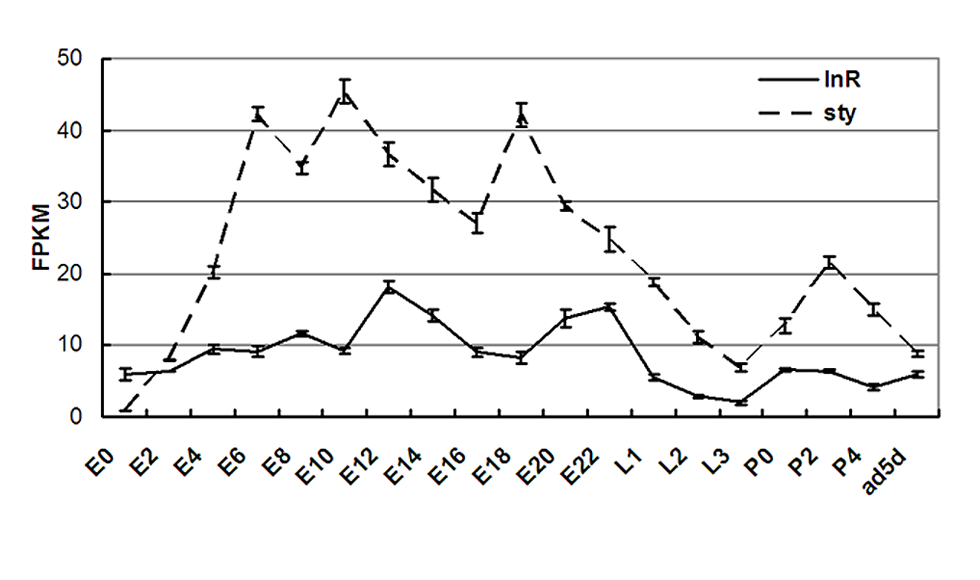

Supplement: Figure S10 — Correlation between the levels of inr and EGFR target sty mRNAs at various stages of Drosophila development. RNA levels were determined using RNA-seq data from modENCODE (www.modencode.org) by cufflinks (cufflinks.cbcb.umd.edu). Spearman's correlation coefficient rho = 0.71 (P<0.001). E# indicates hours of embryonic development. L# indicates larval stage. P# indicates days of pupal development. ad5d indicates 5 day adult. (TIF) [file pgen.1002429.s010.tif]

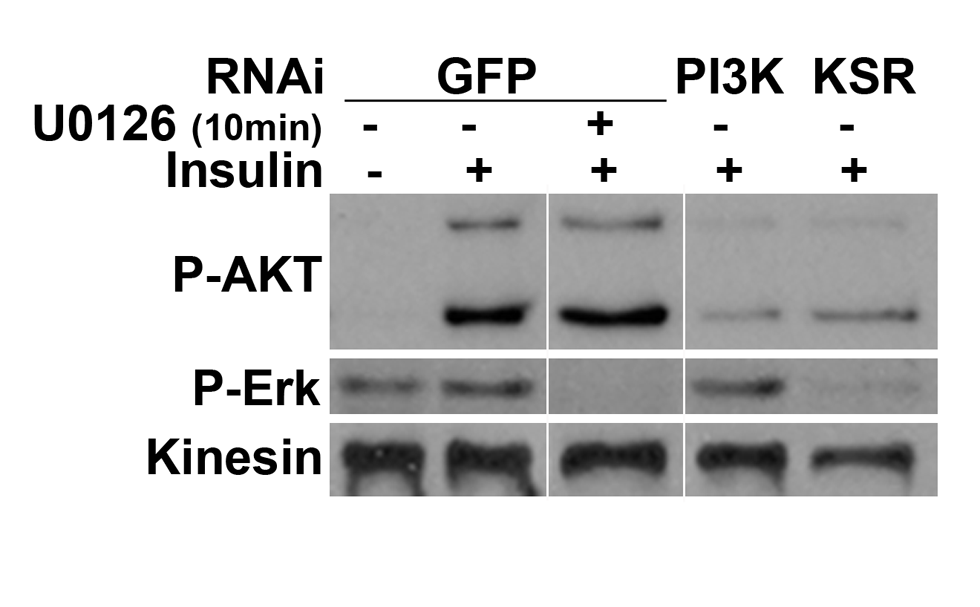

Supplement: Figure S11 — Acute pharmacological inhibition of MAPK/ERK has no impact on insulin activity. Immunoblots to visualize the level of AKT and ERK phosphorylation in S2 cells treated with the MEK inhibitor U0126 (Promega) or in control cells. Cells were treated with dsRNA to deplete PI3K, KSR or GFP as a control and after 5 days stimulated with insulin and treated with 10 µM U0126 for 10 min as indicated. Pharmacological inhibition of MEK by U0126 was effective, as visualized by anti-P-ERK. Anti-Kinesin was used as loading control. Samples were run on the same gel, but intervening lanes have been removed as indicated. (TIF) [file pgen.1002429.s011.tif]
